# Efficacy and safety of low-dose rituximab as induction therapy for antineutrophil cytoplasmic antibody-associated vasculitis with renal involvement: a Chinese case series

**DOI:** 10.1186/s12882-023-03075-8

**Published:** 2023-02-08

**Authors:** Lin Liu, Haitao Lu, Guming Zou, Haifeng Wang, Jing Li, Yue Yang, Jian Zhang, Xueling Wang, Wenge Li, Li Zhuo

**Affiliations:** Department of Nephrology, China-Japan Friendship Hospital, No.2, East Yinghua Street, Beijing, 100029 People’s Republic of China

**Keywords:** Antineutrophil cytoplasmic antibody-associated vasculitis, Rituximab, Low-dose, Renal injury

## Abstract

**Background:**

Rituximab (RTX) is a standard therapy for antineutrophil cytoplasmic antibody (ANCA)-associated vasculitis (AAV). However, the most frequently used dose may lead to severe adverse effects (SAEs). We explored the efficacy and safety of low-dose RTX in Chinese patients with AAV.

**Methods:**

A total of 22 Chinese patients diagnosed with AAV with renal involvement, including 8 treated with low-dose RTX (400 mg of RTX total over 4 weeks) and 14 treated with cyclophosphamide (CYC), were evaluated. The baseline clinical and pathological data and laboratory parameters during follow-up at months 1, 3, 6, and 12 were collected retrospectively.

**Results:**

The baseline data showed no significant differences between the two groups. The median peripheral CD19^+^ cell counts in the RTX group decreased from 315.0/μL to 1.5/μL at 2 weeks, and to 2.5/μL at 1 month after the first dose. The median SCr level decreased from 267.8 μmol/L before treatment to 151.45 μmol/L at 1 month, 132.75 μmol/L at 3 months, 123.2 μmol/L at 6 months, and 151.9 μmol/L at 12 months in RTX-treated patients. The improvements in renal function, proteinuria, and ANCA titre were not significantly different between the two groups. The SAE rate was significantly lower in the RTX group (one SAE of pneumonia) compared with the CYC group.

**Conclusions:**

This is the first report that low-dose RTX could be effective for the treatment of Chinese patients with AAV with renal involvement.

## Background

Antineutrophil cytoplasmic antibody (ANCA)-associated vasculitis (AAV) comprises granulomatosis with polyangiitis (GPA, previously Wegner’s granulomatosis), microscopic polyangiitis (MPA), and eosinophilic granulomatosis with polyangiitis (EGPA, previously Churg-Strauss syndrome). Untreated, MPA and GPA may lead to renal failure within weeks of diagnosis or even be life-threatening, so early aggressive treatment is required. Current induction therapy regimens consist of corticosteroids, immunosuppressive drugs, and plasma exchange (PE). Cyclophosphamide (CYC) is the most frequently used immunosuppressive for induction therapy for AAV. The KDIGO 2021 Clinical Practice Guideline for the Management of Glomerular Diseases recommends rituximab (RTX) as an alternative initial treatment in patients with AAV [1]. The preferred dose is reportedly 375 mg/m^2^ per week for 4 weeks or 1 g at weeks 0 and 2. However, severe adverse reactions such as serious infections and neutropenia can occur, especially in Chinese people [2, 3]. In 27 Chinese patients with AAV treated with RTX, severe infection was reported in 10 patients (37%) during 23.6 ± 14.0 months of follow-up [2]. Among 51 AAV patients with renal involvement treated with RTX at 375 mg/m^2^ × 4, 30 serious adverse events occurred in 21 patients during 18 months of follow-up [3]. Therefore, it would be significant to evaluate low-dose RTX regimens efficacy and safety.

In this study, we retrospectively analysed 8 Chinese patients with AAV on RTX at 100 mg per week for 4 weeks as induction therapy and compared the efficacy and safety with 14 patients with AAV receiving CYC.

## Methods

### Patients

In this retrospective single-center study, records of all patients followed in the Nephrology Department of the China-Japan Friendship Hospital between January 2017 and December 2020 have been reviewed and all the 8 patients fulfilling a diagnosis of AAV with renal involvement treated with low-dose RTX were included. Fourteen patients with AAV with renal involvement treated with CYC in the same hospital were enrolled as controls. All patients met the definition of AAV from the Chapel Hill 2012 Consensus Conference [4]. Renal involvement was defined as evidence of: (1) biopsy-proven active pauci-immune GN; (2) red blood cell casts in urine sediment by microscopy and/or proteinuria; or (3) an increase in serum creatinine (SCr) > 30% (or > 25% decrease in estimated glomerular filtration rate [eGFR]) attributed to AAV [5]. RTX or CYC was used for induction at disease onset and relapse with no contraindications for immunosuppressants such as malignancy or active infection. Inclusion required treatment with RTX or CYC for remission induction without other immunosuppressants and a follow-up period of at least 6 months after the first dose. The study protocol was approved by the Human Ethics Review Committee of the China-Japan Friendship Hospital. Written informed consent was obtained from each patient before performing renal biopsies and AAV-related treatment.

### Treatment

PE (1–2 L) was performed in three patients every other day to a maximum of six times until circulating anti-proteinase-3 (PR3) or anti-myeloperoxidase (MPO) antibodies were undetectable. Methylprednisolone (MP) pulse therapy (500 mg per day) was given for 3 days, followed by prednisone (1 mg/kg per day) for about 1 month. RTX was administered at 100 mg per week for 4 weeks (total 400 mg). Simultaneously, oral prednisolone was reduced to 0.5 mg/kg per day, then tapered at 5 mg per month to a maintenance dose of 5 mg/day as reported in the PEXIVAS study [6]. No further concurrent immunosuppression such as CYC, mycophenolate, or cyclosporine A was used in the eight patients treated with RTX. CYC was given to the controls orally or intravenously with a median cumulative dose of 4.8 g (range 2–9 g). Azathioprine was used for the maintenance regimen for CYC group. Prednisone was started at 1 mg/kg per day and tapered at 5 mg per month to a maintenance dose of 5–10 mg/day in the CYC group.

### Clinical data collection and follow-up

We retrospectively collected the subjects’ clinical and pathological data, including baseline demographic characteristics and laboratory parameters, during follow-up at months 1, 3, 6, and 12. Data analyses comprised standard laboratory tests, including those for blood cell count, SCr, albumin, immunoglobulins, and ANCA titre, plus urinalysis. The ANCA staining pattern was assessed by indirect immunofluorescence, and ANCA specificity for PR3 or MPO was evaluated by an enzyme-linked immunosorbent assay. CD19 was used as a marker for B lymphocytes. Peripheral CD19^+^ B-cell and CD4^+^ T cell counts were determined by flow cytometry (Navios; Beckman Coulter, Brea, CA, USA). B-cell depletion was defined as a CD19^+^ count < 10 cells/μL [7]. CD19^+^ cells were classified into CD19^+^CD5^+^ and CD19^+^CD5^−^ cells by flow cytometry. eGFRs were calculated using the CKD-EPI (Epidemiology Collaboration) formula [8]; and max is the greater of SCr/k or 1). Renal pathology was evaluated based on the Histopathologic Classification of ANCA-Associated Glomerulonephritis (2010) [9]. Patients were classified as focal, crescentic, mixed, or sclerotic [9]. All data were obtained from the same laboratory at China-Japan Friendship Hospital. Adverse effects (AEs)—including acute renal failure, severe infection, cardiac and vascular disorders, leucopoenia, thrombocytopenia, cancer, oedema, pain, fever, and diarrhoea—were monitored during the follow-up period by reviewing the medical records. Severe AEs (SAEs) were defined as AEs requiring hospitalisation.

### Statistical analysis

Statistical analysis was performed using SPSS ver. 23.0 for Windows (IBM Corp., Armonk, NY, USA). Differences in the frequency of variables between groups were analysed by the Fisher exact text and chi-squared test. Continuous data are presented as medians with interquartile range (IQR, 25–75% percentile) unless stated otherwise, and were analysed by the Mann–Whitney U test. Values of *P* < 0.05 were considered to indicate statistical significance.

## Results

### Demographic and clinical characteristics

A total of 22 Chinese patients—8 treated with low-dose RTX (400 mg of RTX total over 4 weeks) and 14 treated with CYC—was included in this study. Most of the patients had the MPO-ANCA phenotype (100% of the RTX group, 92.9% of the CYC group). The clinical characteristics are presented in Tables 1 and 2. RTX was used as first-line induction therapy for onset or relapse in the eight cases (three males, five females), together with 0.5 mg/kg of corticosteroids. The two relapsed cases had received CYC therapy at first onset of AAV 1 and 2 years before this relapse, respectively. No patient had taken other immunosuppressive agents during the follow-up period. In the RTX-treated group, the median SCr level was 267.8 (IQR 167.7–562.6) μmol/L, the median eGFR was 17.7 (IQR 6.5–32.6) mL/min/1.73m^2^, the mean serum albumin level was 27.7 ± 4.4 g/L, and the median 24 h urine protein level was 2.0 (IQR 1.1–3.7) g. Six of the eight patients underwent a renal biopsy, and the median ratio of crescentic glomeruli was 44.5 (IQR 32.0–68.25)%. According to the 2010 Histopathologic Classification of ANCA-Glomerulonephritis [9], three of the six low-dose RTX-treated patients were classified as crescentic, and three as focal. Among the eight patients in the CYC group who underwent a renal biopsy with a qualified specimen (≥ 10 whole glomeruli), five were crescentic and three were focal, with no significant difference compared to the RTX group (*P* > 0.05). Interstitial fibrosis / tubular atrophy (IF/TA) < 25% was seen in all 6 patients in RTX group (100%), and all 8 patients in CYC group (100%).

**Table 1:** Basic data of demographic, clinical and laboratory characteristics of 22 AAV patients treated with RTX or CYC

Characteristics	RTX (*n* = 8)	CYC (*n* = 14)	*P* value
**Patients’ characteristic**			
Age at first treatment (y)	61.5 ± 13.2	60.9 ± 6.6	0.879
Gender(M/F)	3/5	6/8	0.670
**onset / relapse**	6/2	14/0	0.121
**Comorbidities**			
Hypertension, n (%)	3(37.5)	3(21.4)	0.624
Diabetes mellitus	1(12.5)	4(28.6)	0.613
Tobacco	1(12.5)	5(35.7)	0.351
**Lung involvement**, n (%)	3(37.5)	7(50%)	0.675
**Other organs involvement, n(%)**	none	Joints, 3 (21.4%)	0.273
**Laboratory parameters**			
Serum albumin (g/L)	27.7 ± 4.4	28.4 ± 4.3	0.739
Serum creatinine (μmol/L), median (IQR)	267.8 (167.7, 562.6)	297.3 (245.4, 490.7)	0.920
eGFR (ml/min/1.73m^2^), median (IQR)	17.7 (6.5, 32.6)	14.9 (8.7, 19.3)	0.973
24 h urine protein (g), median (IQR)	2.0(1.1, 3.7)	2.1(1.1, 3.4)	0.920
PR3-ANCA/ MPO-ANCA	0/8	1/13	1.000
Immunoglobulin G (g/L), median (IQR)	1555.0 (1090.0, 2117.5)	1490.0 (1051.5, 2220.0)	0.804
Immunoglobulin A (g/L), median (IQR)	255.0 (180.3, 464.3)	304.0 (212.5, 352.5)	0.916
Immunoglobulin M (g/L), median (IQR)	87.5 (59.1, 105.0)	68.2 (53.9, 147.0)	0.916
C3 (mg/dl), median (IQR)	90.9 (62.6, 100.0)	82.7 (68.4, 92.2)	0.595
Hypocomplementemia C3, n (%)	3 (37.5)	3 (21.4)	0.624
C4 (mg/dl), median (IQR)	20.3 (15.6, 30.8)	21.81 (15.2, 24.4)	0.972
Hemoglobin (g/L), median (IQR)	109.5 (101.0, 140.8)	104.5 (96.3, 112.5)	0.165
CD4 + T cells (/μL), median (IQR)	1018.5 (451.25, 1356.25)	616 (500.75, 840.5)	0.238
CD19 + B cells(/μL), median (IQR)	315.0 (204.0, 455.0)	385.5 (112.25, 507.75)	1.000
B1 CD19 + CD5 +	21.0 (8.0, 59.0)	45.5 (9.0, 100.25)	0.628
B2 CD19 + CD5-	288.0 (196.0, 396.0)	332.5 (94.75, 419.75)	0.836
**Histopathologic classification **[9]			
Focal, n (%)	3 (50)	3(37.5)	
Crescentic, n (%)	3 (50)	5(62.5)	
Mixed, n (%)	0	0	
Sclerotic, n(%)	0	0	
IF/TA > 25%, n (%)	6 (100)	8 (100)	
**BVAS**	17.5 ± 4.0	17.0 ± 2.9	0.761
**Main treatments**			
Corticosteroids	8 (100%)	14 (100%)	1.000
MP Pulse (500 mg/d*3d)	1 (12.5%)	5 (35.7%)	0.351
Plasmapheresis			
Number of patients	3 (37.5%)	6 (42.9%)	1.000
Median (range)	5 (5, 5)	6.5 (4.5, 9.25)	0.429
Intravenous immunoglobulin			
Number of patients	3 (37.5%)	4 (28.6%)	1.000

Focal, ≥ 50% normal glomeruli; Crescentic, ≥ 50% glomeruli with cellular crescents; Mixed, < 50% normal, < 50% crescentic, < 50% globally sclerotic glomeruli; Sclerotic, ≥ 50% globally sclerotic glomeruli

**Table 2:** Clinical data before and after RTX of the 8 individual patients

**No**	Age	Sex	Onset or relaps	Lung involvemen	Cr before / after RTX (umol/L)	Alb before / after RTX (g/L)	Urine protein before / after RTX (g/d)	ANCA titer before / after RTX (U/ml)	BVAS before/after RTX	CD19 + B cells Before / 1 months after X (/μL)	**Corticosteroids, PE and IVIG**	**Follow-up period**	**SAEs during the follow-up period**
1	68	F	Onset	No	188.0 /97.7	23.0 / 34.9	8.9 / 0.13	Anti-MPO, 86 / neg	17/0	204/16	P 1 mg/kg before RTX; 0.5 mg/kg from RTX, No PE or IVIG	1 year	none
2	63	F	Onset	No	838.4 / 113.7	25.0 / 44.0	2.7 /0.09	Anti-MPO, 49 / neg	14/0	333/5	P 1 mg/kg before RTX; 0.5 mg/kg from RTX. No PE.IVIG for 20 g*5d, twice	1 year	none
3	67	M	Onset	No	443.9 / 146.7	27.7 / 39.5	1.05 / 0.40	Anti-MPO, 43 / neg	25/12	302/3	P 1 mg/kg before RTX; 0.5 mg/kg from RTX.PE for 5 times.IVIG for 20 g*5d	1 year	none
4	59	M	Relapse	alveolar hemorrhage	182.4 / 295.0	32.7 / 27.7	4.04 / 1.95	Anti-MPO, 55 / 76 (relapsed again at 11 months after RTX)	20/18	137/0	P 10 mg/d. PE for 5 times. No IVIG	1 year	Curable pneumonia at 11 months after RTX
5	75	F	Onset	NO	565.2 / 243.0	30.4 / 45.9	1.37 / 0.286	Anti-MPO, 136, neg	14/12	455/2	P 1 mg/kg before RTX; 0.5 mg/kg from RTX. No PE. IVIG for 20 g*5d	1 year	none
6	70	F	Relapse	NO	162.8 / 157.1	21.0 / 34.0	0.74 / 2.98	Anti-MPO, > 200, neg	18/12	709/2	P 1 mg/kg before RTX; 0.5 mg/kg from RTX. No PE or IVIG	1 year	none
7	32	M	Onset	Pulmonary consolidation	159.3 / 89.3	28.6 / 48.1	2.58 / 0.27	Anti-MPO, 100, 27	19/4	315/3	MP 500 mg*3. P 1 mg/kg before RTX; 0.5 mg/kg from RTX. No PE or IVIG	6 months	none
8	58	F	Onset	Interstitial Pneumonitis	347.7 / 124.5	33.2 / 45.1	1.07 / 0.09	Anti-MPO, 136, neg	13/8	NA/0	P 1 mg/kg before RTX; 0.5 mg/kg from RTX. PE for 6 times. No IVIG	6 months	none

*MP* methylprednisolone, *P* prednisolone, *PE* plasma exchange, *RTX* rituximab, *IVIG* intravenous immunoglobulin. Data after RTX means data at last follow-up at 6 months or 1 year after RTX

Three patients (37.5%) had lung involvement; all received prednisone and one was prescribed MP pulse therapy. Three (37.5%) patients received PE and three (37.5%) received one or two intravenous immunoglobulin (IVIG) administrations. There was no significant difference between the two groups in most laboratory parameters at baseline and in additional treatments (including MP pulse, PE, and IVIG therapy). The clinical data for the RTX group are listed in Table 2.

### Lymphocytes and serum immunoglobulins

**Fig. 1:**
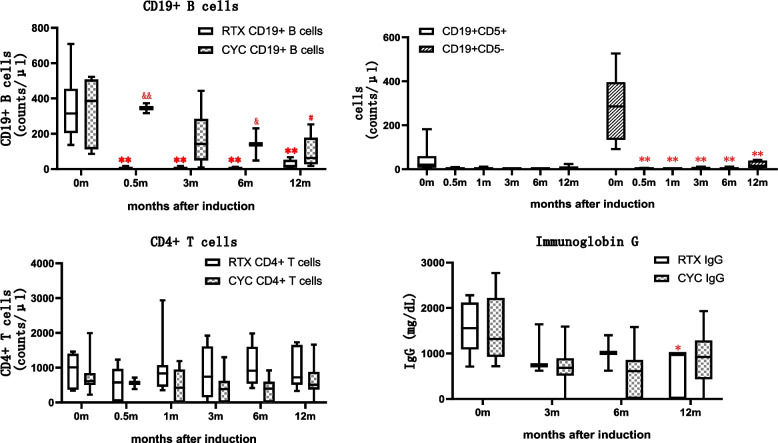
Changes of lymphocytes and IgG in 1 year after RTX or CYC administration. RTX vs baseline: *P < 0.05, **P < 0.01. CYC vs baseline: #*P* < 0.05, ##*P* < 0.01. CYC vs RTX: &*P* < 0.05, &&*P* < 0.01

Before RTX therapy, the median peripheral CD19^+^ cell count in the eight RTX-treated patients was 315.0 (IQR, 204.0–455.0)/μL, similar to patients treated with CYC (Fig. 1). However, CD19^+^ cells were depleted in 83.3% of the patients, in whom the median number decreased to 1.5 (IQR, 1.0–5.75)/μL at 2 weeks after the first 100 mg dose of RTX. Depletion was also noted in 87.5% of the patients to a median count of 2.5 (IQR, 0.5–4.5)/μL at 1 month after induction, and the CD19^+^ cell count remained low for the next 6 months. Only 1 patient did not achieve B cell depletion at 1 month with her CD19 + B cells of 16/μL. At 12 months after RTX administration, the CD19^+^ cell count increased to a median of 17.0 (IQR, 7.5–52.5), significantly lower than at baseline or in the CYC group at 12 months. The CD19^+^CD5^+^ and CD19^+^CD5^−^ counts showed similar trends to the total CD19^+^ cell count. Therefore, low-dose RTX therapy depleted B lymphocytes in the peripheral circulation. The CD4^+^ T-cell count in the RTX group was almost twofold that in the CYC group at 1, 3, 6, and 12 months after induction, although the difference was not significant. The serum IgG level decreased gradually during the follow-up period but was not significantly lower than at baseline until 12 months after RTX administration. Unexpectedly, the decrease in IgG titre was greater in the control group, albeit not significantly so.

### Response and relapse rates

**Fig. 2:**
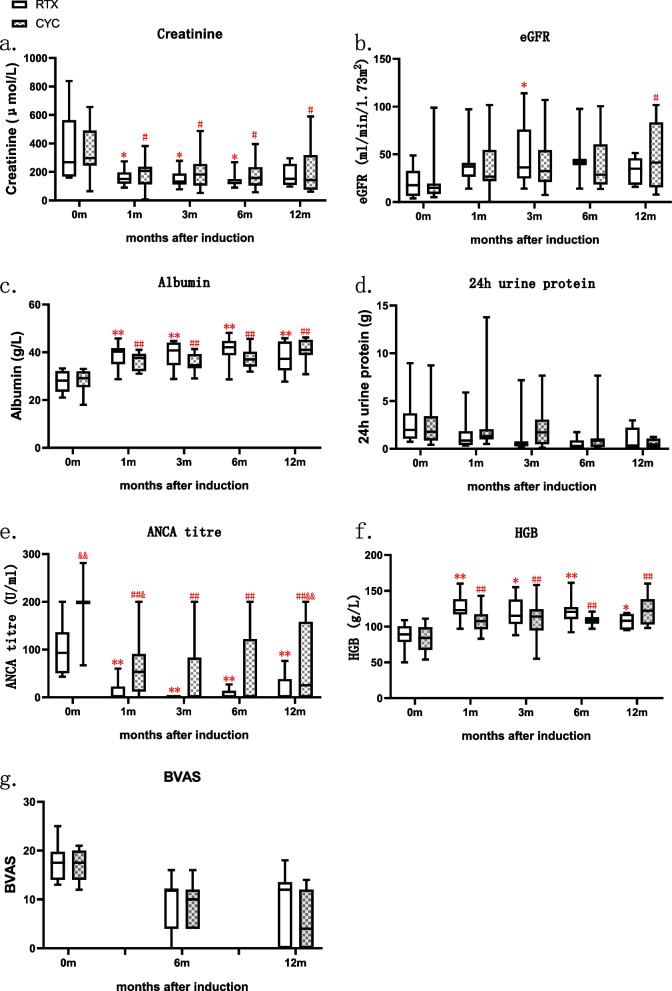
Response to treatment with RTX or CYC in patients with AAV. Changes in serum creatinine (**a**), eGFR (**b**), albumin (**c**), 24 h urine protein (**d**), ANCA titer (**e**), HGB (**f**). BVAS (**g**) RTX vs baseline: *P < 0.05, **P < 0.01. CYC vs baseline: #*P* < 0.05, ##*P* < 0.01. CYC vs RTX: &*P* < 0.05, &&*P* < 0.01

Before any of the induction therapy, the median SCr level was 267.8 (IQR 167.7–562.6) μmol/L with eGFR of 17.7 (IQR 6.5–32.6) ml/min in RTX-treated patients, and 297.3 (IQR, 245.4–490.7) μmol/L with eGFR of 14.9 (IQR 8.7–19.3) ml/min in CYC-treated patients (Fig. 2a–b). RTX was used 1.65 ± 1.26 months later than prednisone and other treatment such as PE and MP pulse, when the SCr level decreased to 165.3 (IQR 117.7–312.5) μmol/L with eGFR of 31.6 (IQR 15.1–41.6) ml/min. CYC was used 1.21 ± 0.77 months later than prednisone, when SCr level of patients in CYC group decreased to 209.9 (IQR 156.9–243.5) μmol/L with their eGFR of 24.1 (IQR 21.7–32.1) ml/min. After RTX therapy, all eight patients showed further improvement in renal function, as indicated by a significant decrease in median SCr to 151.45 (IQR, 118.2–196.0) μmol/L at 1 month. Renal function was stable for the next 6 months, with a median SCr of 132.75 (IQR, 111.6–189.0) μmol/L at 3 months, 123.2 (IQR, 114.4–149.4) μmol/L at 6 months, and 151.9 (IQR, 109.7–256.0) μmol/L at 12 months. During the 12 months after induction, significant improvements in eGFR, serum albumin, and 24 h urine protein were noted in both groups (Fig. 2 a–d). All eight patients had an elevated ANCA titre before RTX induction, which was negative in six patients (75%) at 1 month and seven patients (87.5%) at 6 months after RTX. Although the ANCA titres before RTX were lower than in the CYC group, the decrease in ANCA titre in the RTX group was similar to that in the CYC group during the 12-month follow-up period (Fig. 2e). The hemoglobin level improved significantly in both groups (Fig. 2f). Birmingham Vasculitis Activity Score (BVAS) was lower after treatment in both groups, and no significant difference showed between the two groups (Fig. 2g).

The daily prednisone dose was reduced from ≥ 1 mg/kg/day before RTX therapy to 0.5 mg/kg/day at the initial RTX administration (Table 2) and was gradually reduced thereafter. The prednisone dose was lower in the RTX group than in the control group (1 mg/kg/day) at the time of RTX or CYC administration.

One (#7) of the eight (12.5%) patients experienced a minor relapse, presenting with a slightly elevated ANCA titre without an increase in Cr at 6 months after RTX. Among the six patients followed for 12 months, one (#4) (16.7%) experienced a prominent relapse with an elevated SCr level and re-positive ANCA titre induced by pneumonia at 11 months after RTX administration. The two cases received a second course of RTX. No patient in the CYC group suffered a relapse during the 12-month follow-up period.

### Adverse events

During administration, no patient experienced significant side effects, including an allergy, oedema, paranaesthesia, dysrhythmia, myocardial ischaemia, fever, headache, or joint pain, when 100 mg of RTX was administered intravenously over 4 h. During the follow-up period of 6–12 months, one SAE (curable pneumonia requiring hospitalisation) occurred in the RTX-treated patients (12.5%), and none died (Table 2). The SAE rate was significantly lower in the RTX group than in the CYC group, in which seven patients experienced at least one infection requiring hospitalisation (50%) (*P* = 0.047 [10]), and one (7.1%) died from severe pneumonia.

## Discussion

This is the first report of the effectiveness and side effects of low-dose RTX (100 mg per week for 4 weeks) compared with CYC as an induction regimen for patients with AAV with renal involvement.

A combination of corticosteroids and immunosuppressive agents is the standard therapy for AAV. CYC has been the main immunosuppressive agent used for AAV for more than 30 years. However, it is associated with significant side effects, including bone marrow suppression, infection, haemorrhagic cystitis, secondary amenorrhoea, and cancer, after long-term follow-up [11]. Comorbidities are a major issue in the management of AAV. According to Flossmann, O’s report which recruited 535 AAV patients, infections contributed 48% of deaths within 1 year of induction treatment [12]. The infection rate during induction therapy was 34.7% in patients with AAV. Age at diagnosis, smoking, baseline SCr > 5.74 mg/dL, CD4^+^ T-cell count < 281/μL, and intravenous CYC therapy are risk factors for infection [13]. In this study, patients treated with CYC were observed as control group. The heterogeneity of the cumulative dose of CYC mainly attributed to the side effects in some of the cases treated with CYC.

RTX is a chimeric monoclonal antibody that targets the antigen CD20 on the surface of B cells and clears B cells from the circulation. Compared with traditional immunosuppressive agents, RTX has fewer side effects, including infusion reactions, late-onset neutropenia, and infection [14]. The frequency of infections is related to the cumulative dose of immunosuppressants, concomitant diabetes, and impaired kidney function [15]. The mechanism of RTX in AAV is is not entirely known, but it likely depletes B cells and plasmablasts producing ANCA. However, severe infections were not rare in patients on recommended doses such as four weekly pulses of 375 mg/m^2^ or 1 g twice monthly [14], especially in Chinese patients [2, 16]. Among 27 Chinese AAV patients receiving a mean RTX dose of only 1270 mg, the severe infection rate during the follow-up period of 23.6 ± 14.0 months was 37%, corresponding to an event rate of 20.9 per 100 person-years [2]. RTX at 375 mg × 2 had similar AEs as RTX at 375 mg × 4 in the study by Takakuha et al. [17] Infection occurs in > 50% of patients during the first year after treatment. This led us to hypothesise that the dose used is too high, especially for Chinese patients, who tend to be of relatively small stature.

Low-dose RTX has been successfully used to treat rheumatoid arthritis [18], nephrotic syndrome [19], systemic lupus erythmatosus [20], pemphigus [21], cryoglobulinemia vasculitis [22], and thrombotic thrombocytopenic purpura [23]. A prior study [24] showed that: (1) low-dose RTX could produce significant and durable responses associated with B-cell depletion; (2) the reduced immunosuppressive intensity may result in a more favourable side-effect profile than the standard dose; and (3) low-dose treatment was considerably less costly than standard-dose treatment. Immunosuppression is of concern in AAV because of the advanced age of the patients and the use of high glucocorticoid doses; therefore, the traditional large dose of RTX has a high risk of infection. PE, IVIG, treatment of infections, and prolonged hospitalisation for AAV is costly, to the burden of which is added the high dose of RTX.

Here, we report our single-centre experience of 8 patients receiving low-dose RTX (total, 400 mg) as the immunosuppressive agent in the induction phase, in comparison with 14 concurrent AAV cases on CYC. All patients received oral glucocorticoids, but those on RTX had a higher tapering rate [5]. RTX-treated patients received a lower glucocorticoid dose compared to those treated with CYC, as this showed that the similar outcomes are likely not to be attributed to steroids in the RTX group.

Patients with AAV with renal involvement responded similarly to low-dose RTX and CYC, and there was no significant difference in the increase in eGFR and serum albumin level, or the improvement in 24 h urine protein over the 12-month follow-up period. No patient in the RTX group developed ESRD showing the efficacy of low-dose RTX for inducing the remission of AAV with renal involvement. However, two of eight patients experienced a relapse at 6 and 11 months after RTX therapy, suggesting the need for a second course of RTX treatment at 6 months to maintain remission. As expected, the side-effect rate was low. No patient suffered from infusion reactions, there was one SAE (curable pneumonia requiring hospitalisation) (12.5%), and none of the RTX-treated patients died. The SAE rate was significantly lower in the RTX group than in the CYC group. Therefore, older patients or those susceptible to infection may benefit from low-dose RTX.

This study had several limitations. First, there was heterogeneity in the use of additional treatments (MP pulse, PE, and IVIG therapy) in the RTX and CYC groups; however, the rates of these therapies did not differ significantly between the two groups. Several patients in the CYC group suffered severe side effects, resulting in insufficient cumulative CYC doses (2–9 g). This may have obscured the efficacy of CYC, but it revealed side effects because some patients developed infections after receiving a low dose of CYC. Daily oral and pulse intravenous CYC administration have similar efficacies [25, 26], which attenuated the heterogeneity of our control group. The short follow-up period, small sample size and the lack of a comparison with RTX at usual dose were also limitations. Owing to these limitations, conclusions on low-dose rituximab regimen in subgroups such as patients with ANCA-associated-risk vasculitis (including age > 65, cardiovascular involvement, BVAS > 15, et al.) cannot be drawn. The relapse rate of the RTX group was slightly higher than that of the CYC group, suggesting the need for RTX at the maintenance stage. Therefore, large-scale randomised studies with longer follow-up are required to certify the efficacy and safety of low-dose RTX for AAV with renal involvement.

## Conclusions

This is the first report of the effectiveness and safety of low-dose RTX as induction therapy in Chinese patients with AAV with renal involvement. The improvement in renal injury induced by RTX was similar to that by CYC. The treatment was well tolerated and had a lower complication rate than CYC. This study was limited by its retrospective design and the small number of enrolled patients. This is to our knowledge the first analysis of the efficacy of low-dose RTX for AAV and will facilitate further research on this clinically important issue.

http://www.textcheck.com/certificate/pcNbUR

## Data Availability

All data generated or analyzed during this study are included in this published article.
